# Bayesian Pure-Tone Audiometry Through Active Learning Under Informed Priors

**DOI:** 10.3389/fdgth.2021.723348

**Published:** 2021-08-13

**Authors:** Marco Cox, Bert de Vries

**Affiliations:** ^1^Signal Processing Systems Group, Department of Electrical Engineering, Eindhoven University of Technology, Eindhoven, Netherlands; ^2^GN Hearing, Eindhoven, Netherlands

**Keywords:** active learning, audiometry, Bayesian inference, Gaussian process, machine learning, probabilistic modeling

## Abstract

Pure-tone audiometry—the process of estimating a person's hearing threshold from “audible” and “inaudible” responses to tones of varying frequency and intensity—is the basis for diagnosing and quantifying hearing loss. By taking a probabilistic modeling approach, both optimal tone selection (in terms of expected information gain) and hearing threshold estimation can be derived through Bayesian inference methods. The performance of probabilistic model-based audiometry methods is directly linked to the quality of the underlying model. In recent years, Gaussian process (GP) models have been shown to provide good results in this context. We present methods to improve the efficiency of GP-based audiometry procedures by improving the underlying model. Instead of a single GP, we propose to use a GP mixture model that can be conditioned on side-information about the subject. The underlying idea is that one can typically distinguish between different types of hearing thresholds, enabling a mixture model to better capture the statistical properties of hearing thresholds among a population. Instead of modeling all hearing thresholds by a single GP, a mixture model allows specific types of hearing thresholds to be modeled by independent GP models. Moreover, the mixing coefficients can be conditioned on side-information such as age and gender, capturing the correlations between age, gender, and hearing threshold. We show how a GP mixture model can be optimized for a specific target population by learning the parameters from a data set containing annotated audiograms. We also derive an optimal tone selection method based on greedy information gain maximization, as well as hearing threshold estimation through Bayesian inference. The proposed models are fitted to a data set containing roughly 176 thousand annotated audiograms collected in the Nordic countries. We compare the predictive accuracies of optimized mixture models of varying sizes with that of an optimized single-GP model. The usefulness of the optimized models is tested in audiometry simulations. Simulation results indicate that an optimized GP mixture model can significantly outperform an optimized single-GP model in terms of predictive accuracy, and leads to significant increases the efficiency of the resulting Bayesian audiometry procedure.

## 1. Introduction

Hearing loss is typically represented by an *audiogram*, which depicts the *hearing threshold* (HT) (i.e., the lowest sound intensity level that can still be perceived) at a set of standard frequencies ranging from 125 Hz to 8 kHz. The hearing threshold levels are usually measured through a process called *pure-tone audiometry* (PTA) (1). In PTA, the subject provides a series of “audible” and “inaudible” responses to pure-tones of various frequencies and intensities, and those responses are used to estimate the HT at the required frequencies. Multiple protocols for selecting tone frequencies and intensities have been developed, the most common of which is a staircase “up 5 dB–down 10 dB” approach known as the Hughson-Westlake protocol (2).

We present a method for performing hearing threshold estimation with optimal efficiency in terms of number of interactions required to achieve a given accuracy level. The method is based on a full probabilistic treatment of the estimation problem. At its core is a probabilistic model which captures a range of statistical properties of the hearing threshold. In short, this probabilistic model encodes a probability distribution over hearing thresholds, and describes in a probabilistic way how the subject's response to a stimulus (“audible” or “inaudible”) is generated. Specifically, we propose a model based on a weighted mixture of Gaussian processes (3). Based on this model, our method works in the following way:

1. Optimize the model parameters with respect to a data set of annotated audiometric records from a large number of people. The goal of this step is to train the model to match the statistical distribution of hearing thresholds in the data set as well as possible. This optimization procedure is independent of the subject, and yields a non-personalized prior model.
2. Optionally condition the prior model on side-information from the subject, such as age an gender, to improve the accuracy of the predictive distribution.
3. Determine which stimulus to present such that the “audible” or “inaudible” response to it provides the maximum amount of information about the hearing threshold. Given the model, this stimulus can be derived theoretically using information-theoretic criteria.
4. Present the optimal stimulus to the subject and collect the response.
5. Update the probabilistic model based on the response. This step involves Bayesian inference, and combines the non-personalized model with the data (i.e., the subject's responses to stimuli) to obtain the posterior distribution of the model. The result includes a revised estimate of the hearing threshold including uncertainty bands.
6. Go to step 3 and repeat until the hearing threshold estimate is sufficiently accurate.

The process of repeatedly selecting the most informative next stimulus and updating the probabilistic model based on the response is called an *active learning loop* (4), and it has been shown to significantly reduce the total number of required test tones to reach a certain accuracy level (5). However, the success of the active learning approach hinges on the quality of the probabilistic model at hand. If the model is flexible and accurate enough, it should theoretically outperform any empirical method based on heuristics. If on the other hand the model fails to capture important aspects of the underlying dynamics, the quality of the method will suffer. It is important to note that once the model has been developed and fitted to a data set, the remaining steps 2–6 basically have unique optimal solutions that can be derived theoretically and just have to be translated correctly into an algorithm.

In prior work, the Gaussian process (GP) model has been used to model the hearing threshold as a continuous function of frequency (5–7). The parameters of the GP were typically chosen empirically. In this work we propose the use of a more flexible class of models, namely weighted mixtures of GPs. The rationale behind this is that a lot of hearing thresholds have one of several typical shapes (8). By capturing the statistics of these distinct shapes by separate GP models, the quality of model might be improved significantly. Additionally, the proposed model can be conditioned on side-information such as age and gender of the subject, further increasing the predictive accuracy. The trained and possibly conditioned model can be viewed as an *informed prior* since it is based on information about the target population (through the training set) and the available side-information about the subject (age and/or gender). We show that it is possible to derive theoretical solutions for optimal tone selection and Bayesian model inference under the more flexible model.

The application of Bayesian methods and information-theoretic criteria to obtain information-efficient audiometry procedures has a long history (9–12). Most of the early methods relied on probabilistic models of the HT at individual frequencies, or captured dependencies among a discrete set of frequencies. More recently, GP-based methods with a continuous frequency scale have been introduced (5–7, 13) and validated experimentally (14, 15). The aim of this work is to improve upon those methods by increasing the quality of the underlying model, both by increasing the complexity of the model and by fitting the model to data.

In the remainder, we mathematically specify the proposed model and derive an algorithm to fit its parameters to a set of annotated audiograms. Next, we outline the (approximate) Bayesian inference algorithm required to update the model, as well as the algorithm for selecting the optimal next stimulus. The proposed model is trained on a large data set containing ~176 thousand audiograms annotated with age en gender. The resulting Bayesian PTA method based on an informed prior is tested through various simulations.

## 2. Materials and Equipment

All methods and models have been implemented in the Julia programming language (16). TensorFlow (16) is used as the computational back-end for fitting the hearing threshold models.

### 2.1. Data Source

The results reported in this paper related to model learning and simulations are based on a proprietary data set. This anonymized data set contains the ages, genders, and audiograms of both ears of 88,237 people from the Nordic countries who visited an audiologist. In total, the data set contains 176,474 audiograms annotated with age and gender. The audiograms specify the hearing thresholds with a resolution of 5 dB on (subsets of) the following frequencies: 125, 250, 500, 750, 1,000, 1,500, 2,000, 3,000, 4,000, 6,000, 8,000 Hz. The presented methods are independent of the specific data set that is used for model learning and simulations.

## 3. Methods

We first introduce the probabilistic model on which our method is based. Next, we describe how the model parameters can be “learned” from a data set of annotated audiograms. Subsequently, we show how the model is used to estimate the HT from a set of responses to stimuli by performing Bayesian inference. Finally, we illustrate how the model enables the identification the most informative next stimulus given the responses so far.

### 3.1. Probabilistic Hearing Loss Model

The complete probabilistic model consists of two parts: a user response model and a hearing threshold model. We introduce these components separately, and then combine them to obtain the complete model.

#### 3.1.1. User Response Model

A PTA procedure is assumed to involve a sequence of *trials*. A trial consists of a single pure-tone stimulus of a certain frequency *f* in Hertz and intensity level *h*, together with a binary response label *y* indicating whether the stimulus was audible or inaudible to the subject. The intensity level *h* is expressed in dB *hearing level* (dB-HL), which is a relative sound pressure level in which 0 dB-HL corresponds to the hearing threshold of the average person with no hearing impairment. The subject's response depends on the presented stimulus, and is encoded in the following way:


(1) $$\begin{array}{l} y\left(f,h\right)=\{\begin{array}{ll} +1, & \text{if}\left(f,h\right)\text{is audible},\\ -1, & \text{otherwise}. \end{array} \end{array}$$

A trial is represented by a tuple containing all relevant quantities: (*f, h, y*).

By definition, stimuli near the subject's hearing threshold will not yield consistent responses. To capture the uncertainty in the response generating process, a probabilistic user response model is required. This model describes how a user determines their response to a stimulus if their “true” HT were known. In our model, the “true” HT is assumed to be evaluated under white Gaussian noise $N\left(0,\sigma_{p}^{2}\right)$. In other words, the model assumes that a stimulus is audible if and only if its intensity exceeds the subject's “true” HT at the corresponding frequency by some random margin. This leads to the following formal response model:


(2) $$\begin{array}{l} \begin{array}{l} P\left(y|f,h\right)=\text{Pr}\left\{y\cdot \left(h-\mathrm{HT}\left(f\right)\right)>N\left(0,\sigma_{p}^{2}\right)\right\}\\ \;=\int_{-\infty }^{y\cdot \left(h-\mathrm{HT}\left(f\right)\right)}N\left(h^{\prime }|0,\sigma_{p}^{2}\right)\text{d}h^{\prime }\\ \;=\Phi \left(\frac{y\cdot \left(h-\mathrm{HT}\left(f\right)\right)}{\sigma_{p}}\right), \end{array} \end{array}$$

where HT denotes the unknown “true” hearing threshold as a function of frequency. Φ is the cumulative density function of the standard normal distribution. Since *y* ∈ {−1, +1}, probability distribution *P*(*y* ∣ *f, h*) is a Bernoulli distribution. For simplicity, the perceptual noise variance parameter $\sigma_{p}^{2}$ is independent of frequency, but this can easily be relaxed. The value of $\sigma_{p}^{2}$ can either be learned from a data set of actual pure-tone responses, or it can be tuned empirically.

#### 3.1.2. Hearing Threshold Model

The hearing threshold model specifies a probability distribution over hearing thresholds. Instead of treating hearing thresholds at distinct frequencies as independent quantities, we assume the HT to be a smooth function of frequency. Since the human auditory perception of frequency shifts is non-linear, it makes sense to model the HT in a psycho-acoustical space that resembles the human perception better than the linear frequency domain. Technically, the psycho-acoustical space is a warped frequency space in which the distance between frequencies better resembles the human perception of frequency shifts. This is useful since our model aims to exploit properties of the HT that are more natural to interpret on a psycho-acoustical scale. Various psycho-acoustical scales are being used in the field of audiology, such as the “Mel” scale, the “semitone” scale, and the “Bark” scale (1). All of these scales roughly match the semi-logarithmic frequency axis in typical audiogram plots.

For the HT model it is not very important which specific frequency transformation is used, as long as the transformation is invertible. For the Bark scale, multiple transformations with varying degrees of complexity and accuracy have been proposed (17, 18). In our model we adopt the simple Bark transformation described in (18):


(3) $$\begin{array}{l} \text{bark}\left(f\right)≜6*\sinh^{-1}\left(\frac{f}{600}\right), \end{array}$$

with *f* in Hz. For notational convenience, we will use *x* to denote a transformed frequency and in the remainder of this paper we use HT(x) to denote the HT as a function of the transformed frequency.

We obtain a probabilistic model for hearing thresholds by assuming that a HT is a smooth function of transformed frequency, drawn from a Gaussian process (GP). A GP is a probability distribution over continuous functions, and it is fully characterized by a covariance function and a mean function (3). The approach of modeling the HT by a GP has already been proposed before, for example in (6) and (7). However, instead of assuming the HT to be generated by a single GP, we assume the HT to be generated by one of *C* independently parameterized GPs. The main idea behind this choice is that HTs tend to be of one of several distinct types in terms of location, slope, and smoothness. Mixing multiple GPs has two important benefits. Firstly, it should enable the model to capture the statistical properties of distinct typical HT types with a higher resolution than a single GP, leading to a more accurate model. Secondly, it allows the selection of the individual GP to be dependent on side-information such as age and gender. Intuitively, this means that the individual GP components in the model could capture different HT types corresponding (for example) to mild hearing loss, typical old-age HTs, and “cookie-bite hearing loss.” For example, if the subject's age is available, the model could exploit it by adjusting the a-priori probabilities of the different HT types. For age 80 we would expect the GP corresponding to “typical old-age HTs” to get a higher relative probability compared to age 40. By having a more complex model, we hope to leverage large data sets of annotated audiometric records to be able to *learn* accurate models that capture as many statistical properties as possible.

#### 3.1.3. Complete Probabilistic Model

Our model assumes responses to stimuli to be generated in the following way:

1. Randomly select a GP component *c* ∈ [1, …, *C*] from a categorical distribution whose parameters depend on any available side-information I about the subject: c~Categorical(α(I)).
2. Randomly generate an HT curve from the selected GP: $t\sim \mathrm{GP}\left(m_{c},k_{c}\right)$. Parameters *m*_*c*_ and *k*_*c*_ respectively denote the mean function and covariance function or *kernel* of the GP. The mean function is assumed to be a third-order polynomial, and the covariance function is a squared exponential kernel (3). Note that *t* is a continuous, real-valued function of transformed frequency. The choice for the squared exponential kernel follows prior work (6, 7), and is based on the idea of modeling the HT as a smooth, continuous function of frequency.
3. For each stimulus in the procedure, randomly generate the response based on *t* according to the response model from Equation (2).

I is a set of discrete features, in this case corresponding to the subject's age and gender. The age can be unspecified or an integer between 0 and 120, and gender can be unspecified, female or male.

Formally, this leads to the following generative process for a PTA procedure involving *N* trials on the same subject:


(4a) $$\begin{array}{ll} c & \sim \text{Categorical}\left(\alpha \left(I\right)\right), \end{array}$$

(4b) $$\begin{array}{ll} t|c & \sim \mathrm{GP}\left(m_{c},k_{c}\right), \end{array}$$

(4c) $$\begin{array}{ll} \forall i\in \left[1,\ldots ,N\right]:y_{i}|x_{i},h_{i},t & \sim \text{Bernoulli}\left[\Phi \left(\frac{h_{i}-t\left(x_{i}\right)}{\sigma_{p}}\right)\right]. \end{array}$$

### 3.2. Model Learning

The model from Equation (4) includes a significant number of parameters, specifically:

- *C*: the number of individual GPs in the mixture model.
- α(I): the probabilities of individual GPs as a function on side-information I. Note that α(I) is a vector whose elements sum to 1, and can be interpreted as the conditional mixing weights of the individual GPs.
- *m*_1_, …, *m*_*C*_: the mean functions of the GPs. The mean functions are constrained to be third-order polynomials in transformed frequency space.
- *k*_1_, …, *k*_*C*_: the covariance functions of the GPs, constrained to be squared exponential kernels. A squared exponential kernel is parameterized by a variance parameter and a length-scale parameter. The variance parameter regulates the width of the GP's uncertainty bands around the mean function, and the length-scale parameter regulates the smoothness of the GP. For notational convenience, we use θ_1_, …, θ_*C*_ to denote the parameters of the covariance functions.
- σ_*p*_: the standard deviation of the perceptual noise under which the HT is evaluated.

In general, using a complex model only makes sense if its parameters can be “learned” from a data set. In this context, “learning” means optimizing the parameters such that the model captures the statistics of the data in the data set as well as possible. In other words: “learning” the model implicitly means extracting as much relevant information as possible from the data set, and storing it in the model parameters. We provide an outline for how the model parameters can be “learned” through maximum likelihood estimation.

Since increasing the number of mixture components is guaranteed to lead to a more accurate model (at the cost of more complexity), we propose to optimize *C* empirically. Additionally, σ_*p*_ will also be chosen empirically since it is not possible to learn σ_*p*_ from a data set that consists of (annotated) audiograms without actual responses to individual stimuli. The remaining parameters can be optimized to a data set containing audiograms annotated with optional side-information, for example by using maximum likelihood estimation (MLE). In MLE, the parameters are tuned by maximizing the likelihood that the model assigns to the data.

Assume we have a data set containing audiograms that are optionally annotated with side information. Each audiogram defines the hearing thresholds of a subject's ear at a fixed set of standard audiometric frequencies F, for example F={250,500,750,1,000,1,500,2,000,3,000,4,000,6,000,8,000} Hz. Since the audiograms are only defined at a discrete set of frequencies, the “GP mixture” distribution of infinite dimensionality reduces to a “Gaussian mixture” distribution of finite dimensionality ∥F∥. By exploiting this property, MLE can be performed in three steps:

1. Perform MLE of a Gaussian mixture model (GMM), for example using the well-known expectation-maximization (EM) algorithm (19).
2. Optimize *m*_1_, …, *m*_*C*_ and θ_1_, …, θ_*C*_ by minimizing the Kullback-Leibler divergence between the predictive distributions of the GP mixture and the GMM from step 1. In this step, the parameters of the GP mixture are tuned such that its predictive distribution matches that of the GMM at the discrete set of frequencies F. The optimization can be implemented by writing the Kullback-Leibler divergence in a framework that supports automatic differentiation, and then using the automatically calculated gradients to perform a gradient-based optimization. We implemented this step in TensorFlow (20), and used standard gradient descent to perform the optimization.
3. Implement α(I) by nearest-neighbor regression. For every audiogram that contains side-information, the posterior mixing coefficients are calculated using Bayesian inference, and stored in a lookup table indexed by I, averaging over any duplicate entries. The value of α(I) is then obtained by performing a nearest-neighbor lookup. This approach works well if the data set is large relative to the cardinality of I.

By optimizing the model parameters to a certain data set, the model will assign higher relative probabilities to HT curves that appear more frequently in the data set. This bias has a direct effect on the performance of the HT estimation and optimal stimulus selection methods based on the model. The more representative the data set used for learning is for the population on which the methods are applied, the better the performance will be.

### 3.3. Hearing Threshold Estimation Through Bayesian Inference

Given the (optimized) model and a collection of trials, the HT can be estimated by performing Bayesian inference. In Bayesian inference, the distributions of all random variables in the model are updated to reflect the information in the data, in this case a collection of trials. The result is a posterior probability distribution over the subject's HT curve, including uncertainty bands. Let $D=\left[\left(x_{1},h_{1},y_{1}\right),\ldots ,\left(x_{N},h_{N},y_{N}\right)\right]$ denote a data set containing *N* trials of the same subject. Applying Bayes' rule to the model from Equation (4) yields the following posterior distribution for HT *t*:


(5a) $$\begin{array}{ll} p\left(t|D\right) & \propto p\left(t\right)\cdot p\left(D|t\right) \end{array}$$

(5b) $$\begin{array}{l} =\overset{C}{\underset{c=1}{\sum }}\alpha_{c}\left(I\right)\cdot \mathrm{GP}\left(t|m_{c},k_{c}\right)\cdot p\left(D|t\right) \end{array}$$

(5c) $$\begin{array}{l} =\overset{C}{\underset{c=1}{\sum }}\alpha_{c}\left(I\right)\cdot \frac{1}{A_{c}}\cdot p_{c}\left(t|D\right), \end{array}$$

where $p_{c}\left(t|D\right)$ denotes the posterior distribution of *t* under mixture component *c*, and *A*_*c*_ are scaling factors to satisfy the second equality. This means that the posterior distribution of *t* is a weighted mixture of the posterior distributions of the individual GPs. Thanks to this property, Bayesian inference can be implemented in two steps:

1. Perform Bayesian inference for all *C* mixture components separately, yielding $p_{c}\left(t|D\right)$ and *A*_*c*_.
2. Combine the individual posterior GPs according to Equation (5c).

Under our model, exact Bayesian inference in step 1 is intractable. However, various techniques are available to achieve approximate Bayesian inference, including variational Bayesian inference, Laplace inference and expectation propagation (3). Since a single component from our mixture model resembles a standard GP probit classifier, various GP software libraries can perform the approximate Bayesian inference from step 1 out-of-the-box. A complete derivation of an approximate Bayesian inference algorithm for a single-component using the Laplace method is available in (6).

Using Equation (5c), the posterior distribution of *t* can be written as a weighed sum of the individual posterior GPs:


(6a) $$\begin{array}{ll} p\left(t|D\right) & \propto \overset{C}{\underset{c=1}{\sum }}\alpha_{c}\left(I\right)\cdot \frac{1}{A_{c}}\cdot p_{c}\left(t|D\right) \end{array}$$

(6b) $$\begin{array}{l} =\overset{C}{\underset{c=1}{\sum }}\pi_{c}\cdot p_{c}\left(t|D\right), \end{array}$$

where π_*c*_ is the (unnormalized) posterior mixing weight for component *c*. Once the individual (approximate) posteriors have been obtained, π_*c*_ can be calculated according to:


(7a) $$\begin{array}{ll} \pi_{c} & =\frac{\alpha_{c}\left(I\right)}{A_{c}}, \end{array}$$

(7b) $$\begin{array}{ll} A_{c} & ={\left(\int p\left(D|f\right)p_{c}\left(f\right)\text{d}f\right)}^{-1}. \end{array}$$

*A*_*c*_ is known as the *marginal likelihood* of the data under component *c*, and it is usually returned by the software that implements the approximate inference.

### 3.4. Optimal Stimulus Selection

A central aspect of our Bayesian PTA method is to leverage an optimized probabilistic model of the procedure to repeatedly present the stimulus that will yield the most informative response. Information theory provides a fundamental mathematical framework to enable this. Given a (posterior) probabilistic model, the expected information content of a response about a variable in the model—in our case the HT—can be expressed analytically, allowing the input leading to the response to be optimized with respect to the expected information gain. The approach of actively selecting inputs to trigger responses to learn from is known in the literature as “active learning.” This section provides the derivation of an optimal stimulus selection procedure based on the “Bayesian Active Learning by Disagreement” (BALD) framework from (21).

Given a set of trials D and posterior distribution p(t∣D), the goal is to find the stimulus (x_*_, h_*_) that “maximizes the decrease in expected posterior entropy” of *t* (21):


(8a) $$\begin{array}{ll} \left(x_{*},h_{*}\right) & =\arg\underset{\left(x,h\right)}{\max}\left(\text{H}\left[t|x,h,D\right]-{\text{𝔼}}_{y\sim P\left(y|x,h,D\right)}\text{H}\left[t|y,x,h,D\right]\right). \end{array}$$

In this expression, H[*A*∣*B*] represents the Shannon entropy^1^ of *A* given *B*. In the remainder of this section we work out the objective function under our model. The end result of the derivation can be found in Equation (15).

Inspection of the expression to be maximized reveals that is equal to the mutual information^2^ of the hearing threshold *t* and the binary response *y* to a stimulus (*x, h*) under the posterior distribution: I[t;y∣x,h,D]. Because mutual information is symmetric, *t* and *y* can be exchanged to get an equivalent expression that is easier to evaluate:


(9a) $$\begin{array}{ll} \left(x_{*},h_{*}\right) & =\arg\underset{\left(x,h\right)}{\max}\left(\text{H}\left[t|x,h,D\right]-{\text{𝔼}}_{y\sim P\left(y|x,h,D\right)}\text{H}\left[t|y,x,h\right]\right) \end{array}$$

(9b) $$\begin{array}{l} =\arg\underset{\left(x,h\right)}{\max}\text{I}\left[t;y|x,h,D\right] \end{array}$$

(9c) $$\begin{array}{l} =\arg\underset{\left(x,h\right)}{\max}\text{I}\left[y;t|x,h,D\right] \end{array}$$

(9d) $$\begin{array}{l} =\arg\underset{\left(x,h\right)}{\max}\left(\text{H}\left[y|x,h,D\right]-{\text{𝔼}}_{t\sim p\left(t|D\right)}\text{H}\left[y|t,x,h\right]\right). \end{array}$$

Since *y* is a binary random variable, the entropy terms in Equation (9d) reduce to binary entropy terms^3^.

The first term in Equation (9d) is obtained by writing out the binary entropy of the posterior predictive distribution of *y*, given by:


(10a) $$\begin{array}{ll} P\left(y|x,h;D\right) & =\int P\left(y|t_{x},h\right)p\left(t_{x}|D\right)\text{d}t \end{array}$$

(10b) $$\begin{array}{l} =\int P\left(y|t_{x},h\right)\overset{C}{\underset{c=1}{\sum }}\pi_{c}\cdot p_{c}\left(t_{x}|D\right)\text{d}t \end{array}$$

(10c) $$\begin{array}{l} =\overset{C}{\underset{c=1}{\sum }}\pi_{c}\int P\left(y|t_{x},h\right)p_{c}\left(t_{x}|D\right)\text{d}t, \end{array}$$

where *t*_*x*_ denotes function value *t*(*x*). Since $p_{c}\left(t|D\right)$ is a posterior GP, $p_{c}\left(t_{x}|D\right)$ is approximated by a Gaussian. Let the Gaussian approximate posterior distribution of *t*(*x*) under component *c* be defined as:


(11) $$\begin{array}{l} p_{c}\left(t_{x}|D\right)\approx N\left(t_{x}|\mu_{c},\sigma_{c}^{2}\right), \end{array}$$

where μ_*c*_ and σ_*c*_ are returned by the GP approximate inference engine. Substituting Equation (11) and (4c) in Equation (10) and evaluating the integral yields^4^:


(12a) $$\begin{array}{ll} P\left(y|x,h;D\right) & \approx \overset{C}{\underset{c=1}{\sum }}\pi_{c}\int \Phi \left(\frac{y\cdot \left(h-t_{x}\right)}{\sigma_{p}}\right)\cdot N\left(t_{x}|\mu_{c},\sigma_{c}^{2}\right)\text{d}t_{x} \end{array}$$

(12b) $$\begin{array}{l} =\overset{C}{\underset{c=1}{\sum }}\pi_{c}\Phi \left(\frac{y\cdot \left(h-\mu_{c}\right)}{\sqrt{\sigma_{p}^{2}+\sigma_{c}^{2}}}\right). \end{array}$$

With this, the first term in the objective function from Equation (9d) resolves to:


(13a) $$\begin{array}{l} \text{H}\left[y|x,h,D\right]\approx \text{h}\left(\overset{C}{\underset{c=1}{\sum }}\pi_{c}\Phi \left(\frac{h-\mu_{c}}{\sqrt{\sigma_{p}^{2}+\sigma_{c}^{2}}}\right)\right). \end{array}$$

The second term in Equation (9d) is intractable but can be approximated very well by replacing the binary entropy function by a squared exponential function as proposed in (21):


(14) $$\begin{array}{l} \begin{array}{l} {\text{𝔼}}_{t\sim p\left(t|D\right)}\text{h}\left[y|t,x,h\right]\approx \int \text{h}\left(\Phi \left(\frac{h-t_{x}}{\sigma_{p}}\right)\right)\overset{C}{\underset{c=1}{\sum }}\pi_{c}N\left(t_{x}|\mu_{c},\sigma_{c}^{2}\right)\text{d}t_{x}\\ \;=\overset{C}{\underset{c=1}{\sum }}\pi_{c}\int \text{h}\left(\Phi \left(\frac{h-t_{x}}{\sigma_{p}}\right)\right)N\left(t_{x}|\mu_{c},\sigma_{c}^{2}\right)\text{d}t_{x}\\ \;\approx \overset{C}{\underset{c=1}{\sum }}\pi_{c}\int \exp\left(-\frac{{\left(h-t_{x}\right)}^{2}}{\sigma_{p}^{2}\pi \ln\;2}\right)N\left(t_{x}|\mu_{c},\sigma_{c}^{2}\right)\text{d}t_{x}\\ \;=\overset{C}{\underset{c=1}{\sum }}\frac{\pi_{c}K}{\sqrt{\sigma_{c}^{2}+K^{2}}}\exp\left(\frac{-{\left(h-\mu_{c}\right)}^{2}}{2\left(\sigma_{x}^{2}+K^{2}\right)}\right), \end{array} \end{array}$$

with $K=\sigma_{p}\sqrt{\frac{\pi \ln\;2}{2}}$. With this, the expression for the most informative stimulus from Equation (9d) evaluates to:


(15) $$\begin{array}{l} \left(x_{*},h_{*}\right)=\arg\underset{\left(x,h\right)}{\max}\text{h}\left(\overset{C}{\underset{c=1}{\sum }}\pi_{c}\Phi \left(\frac{h-\mu_{c}}{\sqrt{\sigma_{p}^{2}+\sigma_{c}^{2}}}\right)\right)\\ \;-\overset{C}{\underset{c=1}{\sum }}\frac{\pi_{c}K}{\sqrt{\sigma_{c}^{2}+K^{2}}}\exp\left(\frac{-{\left(h-\mu_{c}\right)}^{2}}{2\left(\sigma_{x}^{2}+K^{2}\right)}\right). \end{array}$$

The most straightforward way to find (x_*_, h_*_) is to perform an (adaptive) grid search in the desired feasible set, or to use another global optimization method. Since the objective in Equation (15) is relatively cheap to evaluate in terms of computations, this is no problem in practice. However, it is also possible to perform the optimization in a cheaper way by adding an additional approximation. Instead of using the Gaussian mixture posterior directly to obtain the objective function, one could first approximate the mixture posterior by a single Gaussian posterior through moment matching. Plugging this single Gaussian approximate posterior into the objective function eliminates the sums in Equation (15). In that case, x_*_ turns out to correspond to the transformed frequency on which the approximate posterior has the largest variance, which can be found using a one-dimensional grid search. Once x_*_ is known, h_*_ is equal to the posterior mean of the HT at x_*_ under the single Gaussian approximation (6). It is also possible to only use of the single Gaussian approximation to find x_*_, and then solve for h_*_ under the full model using a simple line search.

## 4. Results

### 4.1. Model Learning

To evaluate the predictive performance of the probabilistic model, we learned multiple models with the numbers of mixture components ranging from 1 to 10. All models are learned from the same training set. Training and test sets are obtained by randomly splitting the data set described in section 2.1 subject-wise, such that the training set contains the data of 80% of the subjects (70,590 subjects, 141,180 audiograms) while the test set covers the remaining 20% of subjects.

The quality of a probabilistic model is determined by the extent to which it can predict data in the test set. The better the model has captured the properties and statistics of the data set, the higher the (average) probabilities it assigns to records in the test set. The predictive accuracy of a model is typically measured by the average posterior log-likelihood of records in the test set. The posterior log-likelihood of a hearing threshold *t* specified at a set of transformed frequencies X is defined as:


(16) $$\begin{array}{l} \log p\left(t|D\right)=\underset{x\in X}{\sum }\log p\left(t_{x}|D\right), \end{array}$$

where $p\left(t_{x}|D\right)$ is the value of the posterior distribution of the hearing threshold evaluated at transformed frequency *x*.

**Figure 1:**
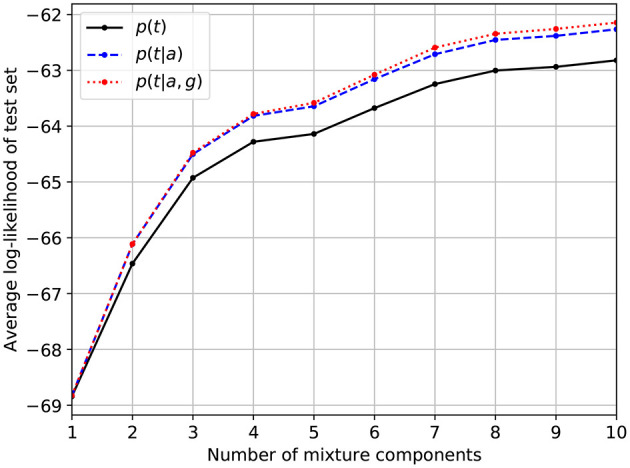
Predictive accuracy of the optimized models as measured by the average log-likelihood of audiograms in the test set. The dashed and dotted lines denote the effect of conditioning the model on age (*a*) and/or gender (*g*).

Figure 1 shows the average log-likelihood of records in the test set as a function of the number of mixture components. Separate lines are used to show the effect of conditioning the models on age and/or gender. As is to be expected, increasing the number of mixture components monotonically increases the predictive accuracy, although the incremental benefit of extra components diminishes after about seven components. Conditioning on age or gender has a positive effect on performance if the number of mixture components is sufficiently large. Conditioning on age has a stronger impact than conditioning on gender.

**Figure 2:**
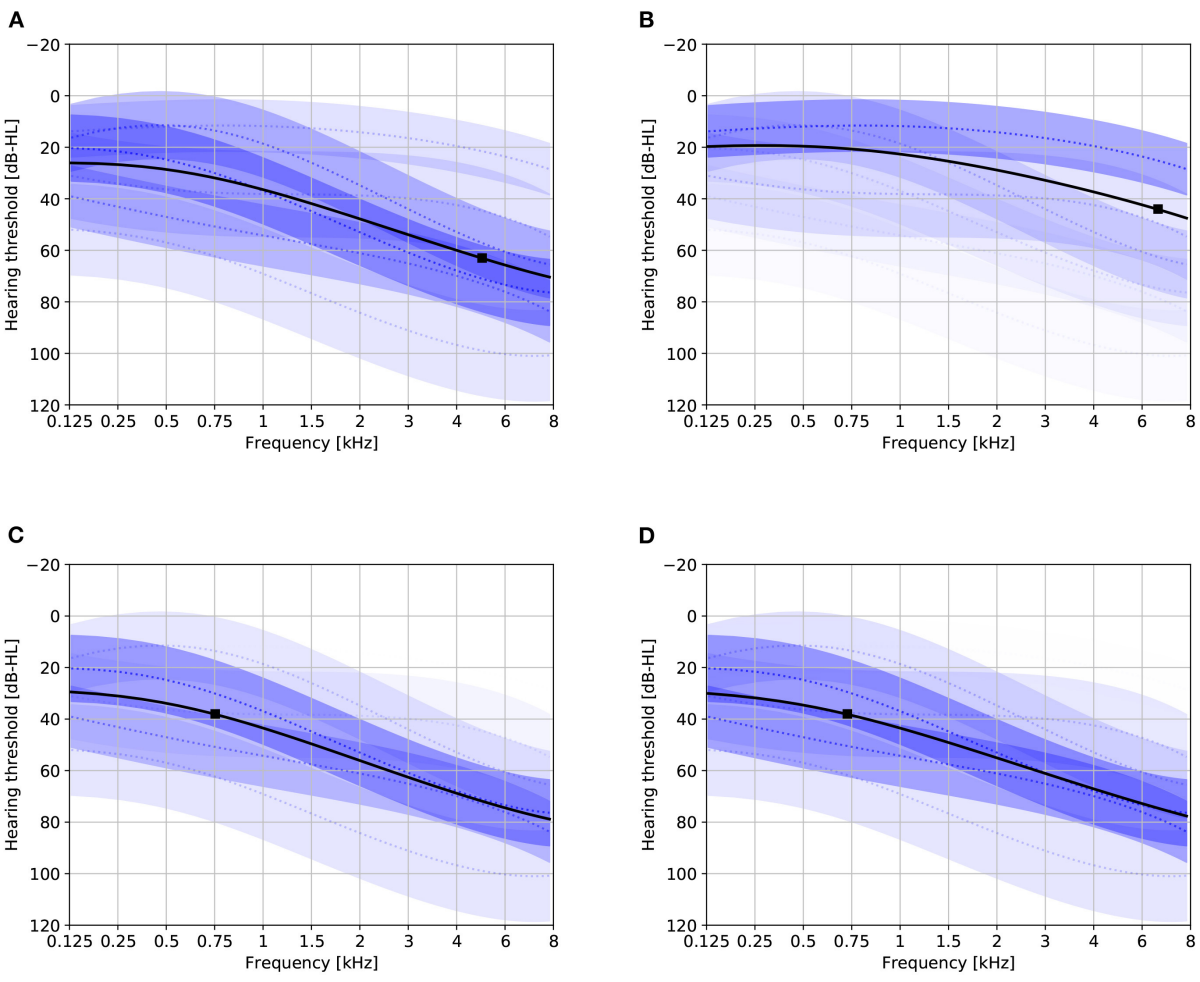
Audiogram plots visualizing the learned model containing six mixture components with various conditionings. Solid black lines denote the initial HT estimate. Dotted blue lines and shaded areas depict the individual GP mixture components ±1 standard deviation (the transparency is proportional to the mixing weight). Solid black boxes indicate the optimal first stimulus. **(A)** Not conditioned on age or gender. **(B)** Age 40, gender unspecified. **(C)** Age 80, gender unspecified. **(D)** Age 80, female.

**Figure 3:**
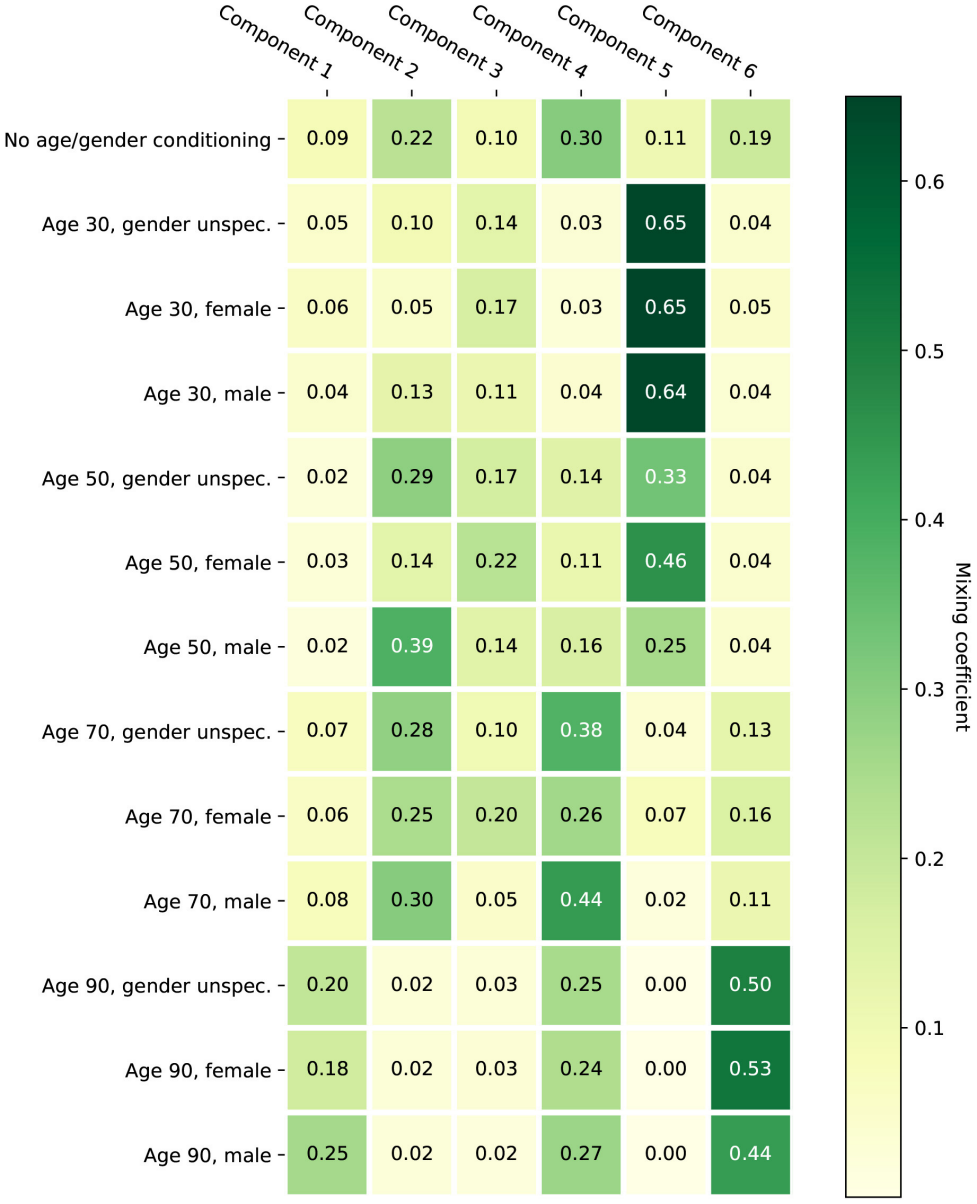
A-priori mixing weights of the learned six-component model under various conditionings.

Figure 2 provides a visualization of the learned model with six mixture components under various conditionings. Without conditioning on age and gender, the distribution of the HT should be representative for the complete population represented by the training set. The effect of conditioning on age is clearly visible: for age 40 the mixture components corresponding to mild hearing loss get assigned a higher weight compared to age 80. The effect of conditioning on gender is smaller. The plots also show that the first proposed stimulus can be different based on age and gender. Figure 3 shows the a-priori component mixing weights of the six-component model under various conditionings, providing a visual overview of the relative importance of the different components per age and gender group.

**Figure 4:**
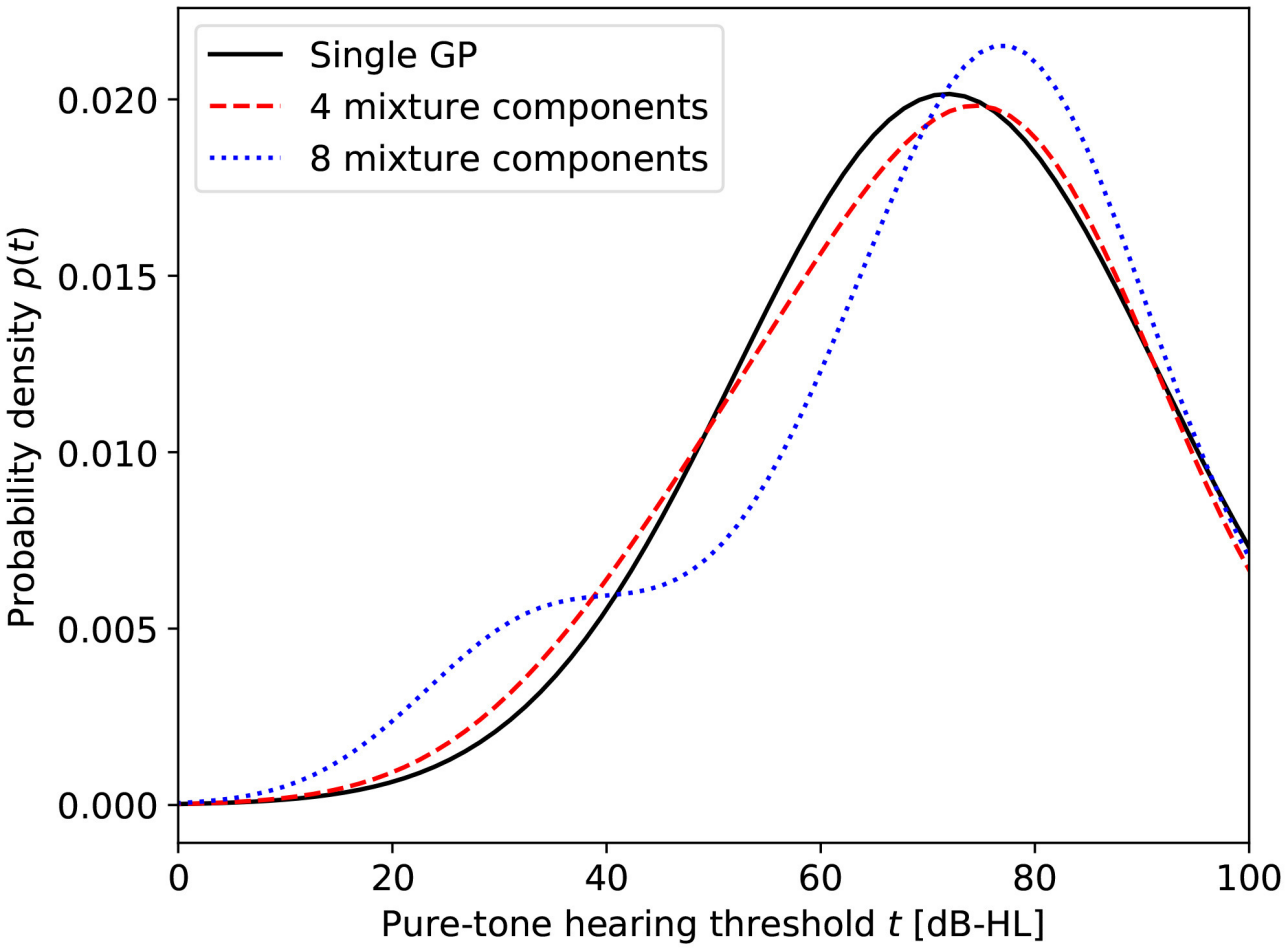
Probability distributions of the HT at 8 kHz for different numbers of mixture components.

To inspect the effect of using a mixture of GPs instead of a single GP, we evaluated the posterior probability distributions of the hearing threshold at fixed frequencies under the various models. Figure 4 shows these probability distributions at 8 kHz for a selected number of models. The figure clearly illustrates the increased ability of the model to capture the non-Gaussian distribution of the hearing threshold as the number of mixture components is increased.

### 4.2. Bayesian PTA Simulations

To test the usefulness of our method, we performed PTA simulations on a random subset of 200 audiograms from the test set. Identical simulations are performed using both the learned single-component model and the learned 8-component model, which seems to provide a good trade-off between predictive accuracy and computational complexity judging from Figure 1. For each of the 200 randomly selected audiograms, a PTA simulation is performed in the following way.

1. Interpolate the HT in the audiogram (which is only defined on a subset of the standard audiometric frequencies) linearly, yielding a piecewise linear function of frequency.
2. Optionally condition the model on the age and gender corresponding to the audiogram.
3. Determine the optimal next stimulus according to Equation (9d).
4. Simulate the response based on the (interpolated) HT and the response model from Equation (2). Update the model with the response by performing Bayesian inference.
5. Go to step 3 and repeat until 25 responses have been collected.

**Figure 5:**
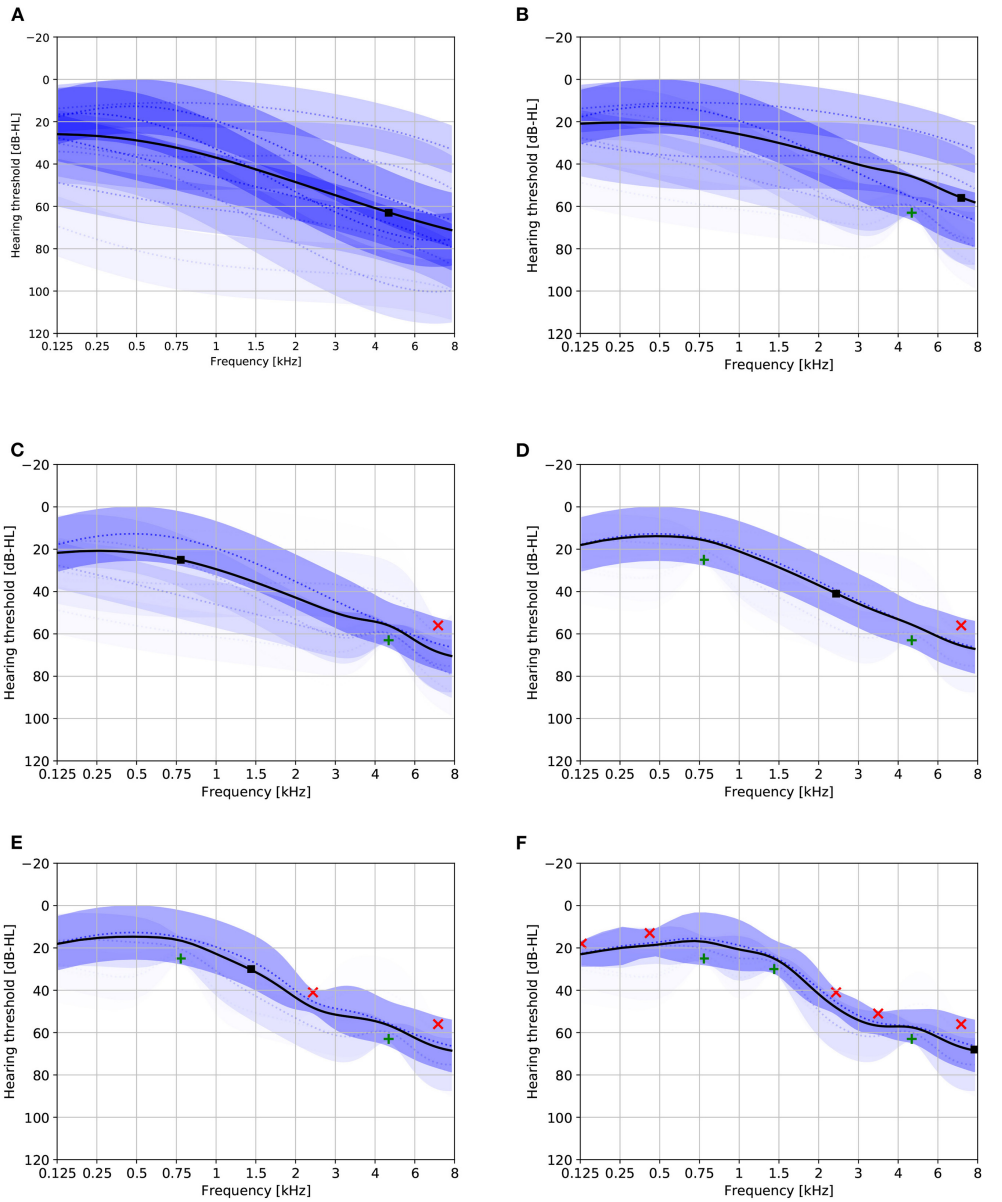
Audiogram plots visualizing the progression of a single Bayesian PTA simulation under the eight-component model. Solid black lines denote the HT estimate. Dotted blue lines and shaded areas depict the individual GP mixture components ±1 standard deviation (the transparency is proportional to the mixing weight). Green and red crosses, respectively, depict audible and non-audible responses. Solid black boxes indicate the optimal next stimulus. **(A)** Audiogram based on zero responses. **(B)** Audiogram based on one response. **(C)** Audiogram based on two responses. **(D)** Audiogram based on three responses. **(E)** Audiogram based on four responses. **(F)** Audiogram based on eight responses.

Figure 5 provides a visualization of the progression of a single Bayesian PTA simulation under the 8-component model. A couple of observations can be made from this figure:

- The posterior mixing weights tend to converge toward either 0 or 1 as more responses are incorporated. Intuitively, this can be interpreted as the model first detecting which mixture component best matches the responses, and then refining the estimate based on the most dominant mixture component. It is this ability that enables the mixture model to attain a faster convergence rate than a single GP model.
- The overall uncertainty about the HT as measured by the variance of the HT estimate tends to decrease as more responses are incorporated.
- The optimal stimuli proposed by the method seem to approximate a randomized grid search in the frequency dimension.

**Figure 6:**
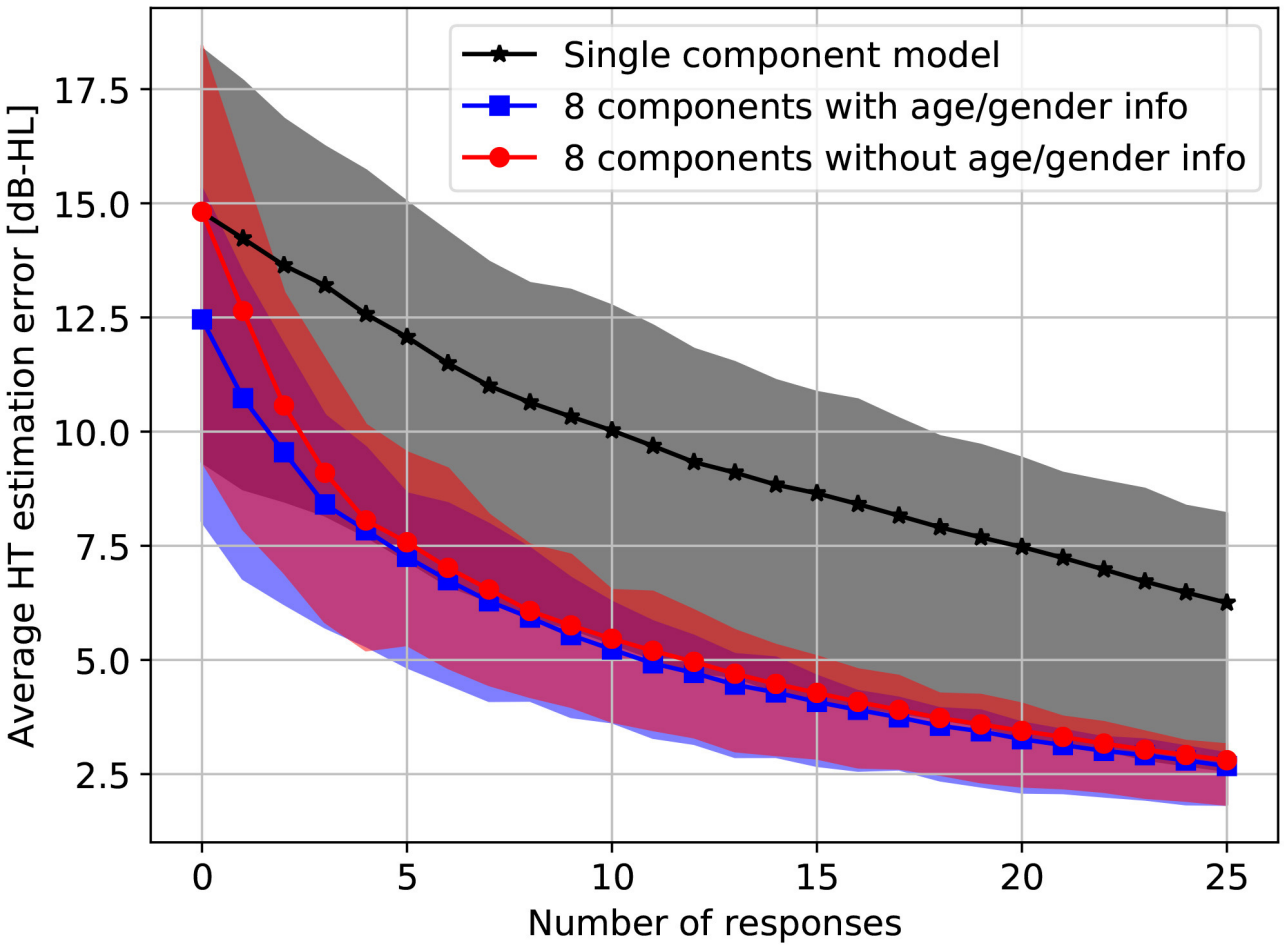
Average absolute HT estimation error on the standard audiometric frequencies (125, 250, 500, 750, 1,000, 1,500, 2,000, 3,000, 4,000, 6,000, 8,000 Hz) as a function of the number of responses under the single-component and 8-component models. The results are averaged over 200 simulations on a random subset of the test set. Shaded areas span from the first quartile to the third quartile. Note that the single-component model cannot be conditioned on age or gender.

To quantify the performance of our methods in the simulations, we calculate the average absolute error of the HT estimates after every simulated response. The average absolute estimation error is obtained by averaging the absolute differences between the assumed HT and the posterior HT estimate at the following frequencies: 125, 250, 500, 750, 1,000, 1,500, 2,000, 3,000, 4,000, 6,000, 8,000 Hz. Figure 6 shows the evolution of the estimation error as responses are added. Both the single-component model and the 8-component model result in a monotonic decrease in estimation error, although the decrease is significantly faster under the 8-component model. Using age and gender information consistently decreases the estimation error, with the effect being largest before any responses have been processed. As more responses are processed, the relative benefit of age and gender information diminishes.

## 5. Discussion

We have demonstrated how a practical and data-efficient PTA method can be obtained by taking a probabilistic modeling approach. Any PTA method involves at least two parts: a method to select stimuli and a prescription for estimating the HT based on responses to said stimuli. Instead of defining these parts in a direct way, we have shown that if one starts with a probabilistic model of the response-generating process, both parts can be derived in a natural way based on formally defined objectives. In the case of stimulus selection, the concept of expected information gain can be used to derive a method that sequentially selects optimal stimuli (in terms of information retrieval rate about the HT) under a given model. The task of estimating the HT based on responses reduces to one of Bayesian posterior inference under the probabilistic modeling approach. Since both parts arise naturally given the underlying model, the model specification indirectly specifies the complete PTA method. As a result, improvements in the quality of the underlying model directly translate into an improved PTA method, either in terms of estimate convergence rate or robustness. Combining a probabilistic modeling approach with information gain maximization in the context of audiometry already has a long history (9, 10, 12). More recently, such approaches have been extended based on GP models (5–7, 13). Various studies have been conducted to experimentally validate these methods (14, 15).

The focus of this work was to increase the efficiency and accuracy of GP-based PTA methods by improving the quality of the underlying model. Toward this end, we proposed a more complex model, i.e., a finite mixture of GPs with mixing weights that depend on additional information about the subject. Moreover, we leverage data to optimize the parameters of the more complex model for a specific target population. The combination of a more complex model and a data-driven optimization leads to a model of higher quality, while preserving the ability to derive optimal stimulus selection and HT estimation based on Bayesian inference. Our simulations indicate that the improved model indeed yields a PTA method with a significantly faster convergence rate than that of an optimized single-GP model. The ability of the proposed model to be conditioned on age and gender also increases performance. The increased performance comes at the price of increased computational complexity. The computational complexity of the Bayesian inference algorithm is linear in terms of the number of mixture components. The same holds for the complexity of the optimal trial selection algorithm if the described approximation is applied. As a result, the computational complexity of our method with a mixture of *K* components requires roughly *K* times the amount of computations required under a single-GP model.

We identify multiple possible directions to improve upon the described methods. Firstly, making the user response model (i.e., the part of the model that specifies how a response to a stimulus is generated given the HT) dependent on frequency should increase the quality of the model, given the likeliness that such a relation indeed exists. Ideally, the parameters of a more complex user response model should be learned from data as well, which would require a data set containing raw audiometric test data. Secondly, the method could be made more robust to corrupted responses. In practice, it is to be expected that responses are sometimes inverted by accident, for example due to external disturbances, mistakes, or hardware malfunction. If corrupted responses occur, an optimal active learning method will have a hard time recovering unless the underlying model explicitly incorporates a data corruption aspect. Extending the model with a data corruption part will increase the robustness at the expense of slower convergence, since the assumed signal-to-noise ratio of the responses will decrease. Another option would be to try to detect corrupted responses post hoc, and then excluding them from the data. A third possible improvement is to exploit the correlation between the HTs at both ears of the same subject. Preliminary analysis of our data set indicates that there is a statistically significant correlation to be exploited. One could extend the model such that the result of the PTA procedure on the one ear could be used to improve the predictive accuracy about the HT at the other, leading to a speedup of the PTA procedure at the second ear.

## Data Availability Statement

The data analyzed in this study is subject to the following licenses/restrictions: The data set is proprietary. Requests to access these datasets should be directed to Bert de Vries, bert.de.vries@tue.nl.

## Author Contributions

MC and BV jointly developed the described methods. MC wrote the software implementation of the methods, implemented and executed the experiments, and wrote the manuscript. BV reviewed and revised the manuscript. Both authors contributed to the article and approved the submitted version.

## Conflict of Interest

The authors declare that the research was conducted in the absence of any commercial or financial relationships that could be construed as a potential conflict of interest.

## Publisher's Note

All claims expressed in this article are solely those of the authors and do not necessarily represent those of their affiliated organizations, or those of the publisher, the editors and the reviewers. Any product that may be evaluated in this article, or claim that may be made by its manufacturer, is not guaranteed or endorsed by the publisher.

## Footnotes

^1^Shannon entropy is a measure of uncertainty about the value of a random variable: H[*A*] ≜ 𝔼[−log*p*(*A*)]. A conditional entropy corresponds to the entropy of a conditional probability distribution: H[*A*∣*B*] ≜ 𝔼[−log*p*(*A*∣*B*)].

^2^Mutual information is a symmetrical measure of dependence between two random variables, which can be expressed in terms of (conditional) entropies: I[*A*; *B*] = I[*B*; *A*] = H[*A*]−H[*A*∣*B*].

^3^Binary entropy function h(·) relates the odds parameter of a binary random variable to its Shannon entropy: *x*~Bernoulli(α)⇒H[*x*] = h(*x*) ≜ −αlog(α)−(1−α)log(1−α).

^4^A derivation of the solution to the integral can be found in section 3.9 of (3).
